# Development of a Dengue Virus Serotype-Specific Non-Structural Protein 1 Capture Immunochromatography Method

**DOI:** 10.3390/s21237809

**Published:** 2021-11-24

**Authors:** Kanaporn Poltep, Emi E. Nakayama, Tadahiro Sasaki, Takeshi Kurosu, Yoshiki Takashima, Juthamas Phadungsombat, Nathamon Kosoltanapiwat, Borimas Hanboonkunupakarn, Sarin Suwanpakdee, Hisham A. Imad, Narinee Srimark, Chiaki Kitamura, Atsushi Yamanaka, Akio Okubo, Tatsuo Shioda, Pornsawan Leaungwutiwong

**Affiliations:** 1 Department of Microbiology and Immunology, Faculty of Tropical Medicine, Mahidol University, Bangkok 10400, Thailand; kanaporn.pol@gmail.com (K.P.); nathamon.kos@mahidol.ac.th (N.K.); pornsawan.lea@mahidol.ac.th (P.L.); 2 Mahidol-Osaka Center for Infectious Diseases (MOCID), Faculty of Tropical Medicine, Mahidol University, Bangkok 10400, Thailand; emien@biken.osaka-u.ac.jp (E.E.N.); juthamas@biken.osaka-u.ac.jp (J.P.); imad@biken.osaka-u.ac.jp (H.A.I.); nsrimark@gmail.com (N.S.); knmya@biken.osaka-u.ac.jp (A.Y.); 3 The Monitoring and Surveillance Center for Zoonotic Diseases in Wildlife and Exotic Animals, Faculty of Veterinary Science, Mahidol University, Nakhon Pathom 73170, Thailand; sarin.suw@mahidol.edu; 4 Department of Viral Infections, Research Institute for Microbial Diseases (RIMD), Osaka University, Osaka 565-0871, Japan; sasatada@biken.osaka-u.ac.jp; 5 Center for Infectious Disease Education and Research, Osaka University (CiDER), Osaka 565-0871, Japan; 6 ARKRAY, Inc., Yousuien-nai, 59 Gansuin-cho, Kamigyo-ku, Kyoto 602-0008, Japan; takashimay@arkray.co.jp (Y.T.); kitamura.td@arkray.co.jp (C.K.); okubo@arkray.co.jp (A.O.); 7 Department of Virology I, National Institute for Infectious Diseases, Tokyo 208-0011, Japan; kurosu@niid.go.jp; 8 Department of Clinical Tropical Medicine, Faculty of Tropical Medicine, Mahidol University, Bangkok 10400, Thailand; borimas.han@mahidol.edu

**Keywords:** dengue virus, serotype-discrimination, rapid test, immunochromatography, NS1

## Abstract

Four serotypes of dengue virus (DENV), type 1 to 4 (DENV-1 to DENV-4), exhibit approximately 25–40% of the difference in the encoded amino acid residues of viral proteins. Reverse transcription of RNA extracted from specimens followed by PCR amplification is the current standard method of DENV serotype determination. However, since this method is time-consuming, rapid detection systems are desirable. We established several mouse monoclonal antibodies directed against DENV non-structural protein 1 and integrated them into rapid DENV detection systems. We successfully developed serotype-specific immunochromatography systems for all four DENV serotypes. Each system can detect 10^4^ copies/mL in 15 min using laboratory and clinical isolates of DENV. No cross-reaction between DENV serotypes was observed in these DENV isolates. We also confirmed that there was no cross-reaction with chikungunya, Japanese encephalitis, Sindbis, and Zika viruses. Evaluation of these systems using serum from DENV-infected individuals indicated a serotype specificity of almost 100%. These assay systems could accelerate both DENV infection diagnosis and epidemiologic studies in DENV-endemic areas.

## 1. Introduction

Dengue is a serious disease of public importance, with increasing worldwide spread [1]. The disease is caused by infection with the dengue virus (DENV). Since the first isolation of DENV in 1943, four antigenically distinct serotypes (DENV-1–4) have been identified. Global phenomena such as urbanization and increased international travel are key factors facilitating the spread of dengue. Documenting the type-specific spread of DENV could have important implications in understanding patterns in dengue hyperendemicity and disease severity, as well as vaccine design and deployment strategies [2]. Some reports suggest that disease severity varies among DENV serotypes [3].

Identifying the DENV serotype is important for two primary reasons: (i) risk factors for severe dengue have been associated with more pathogenic DENV serotypes; and (ii) the involvement of different DENV serotypes in primary and secondary infections is believed to be a risk factor for severe disease [4]. This is probably due to the targeting of virus binding with non-neutralizing antibodies to macrophages via interaction with the Fcγ-receptor, thereby increasing virus transmission, a phenomenon known as antibody-dependent enhancement (ADE) [5,6]. Primary infection leads to the secretion of serotype-specific antibodies that neutralize viruses of that serotype. In contrast, antibodies that cross-react with a virus of a different serotype in secondary infection may not neutralize the virus and instead enable the virus to bind to monocytic cells.

In addition to the increased severity of secondary infection, certain DENV serotypes are associated with more severe clinical manifestations, even during primary infection. Yung et al. (2015) reported that in Singapore, where DENV-1 and DENV-2 are major circulating serotypes, DENV-1 is associated with both dengue hemorrhagic fever (DHF) and severe dengue, whereas DENV-2 has a lower risk of DHF. In Brazil, patients of the 2010 epidemic caused by DENV-2 had lower platelet counts than patients of the 2013 epidemic caused by DENV-4, in which secondary infections involved both DENV-2 and DENV-4 [7,8]. No severe dengue cases were reported in Argentina during the 2014 DENV-4 outbreak [9], although half of detected infections were secondary. However, one report from Brazil claimed 44 deaths occurred in the 2012 DENV-4 outbreak [10]. In Thailand, secondary infection with DENV-2 was associated with DHF [11,12], whereas primary infection with DENV-1 was found in hospitalized children [13]. Vicente et al. (2016) reported that between 2009 and 2013 in Brazil severe dengue was 7 times more frequent among patients infected with DENV-2 than among patients infected with other serotypes [14]. Individuals infected with DENV-3 had a higher prevalence of musculoskeletal and gastrointestinal manifestations, and a higher prevalence of respiratory and cutaneous manifestations was observed in individuals with febrile disease infected with DENV-4 in Peru, Bolivia, Ecuador, and Paraguay between 2005 and 2010 [15]. In Cuba, 16% of primary infections with DENV-3 reportedly became symptomatic, whereas virtually all individuals with secondary DENV-3 infection 23–24 years after the primary infection with DENV-1 became symptomatic, and 3% developed DHF. In contrast, only 20% of individuals with secondary DENV-2 infection 20 years after DENV-1 primary infection became symptomatic, with no reported cases of DHF [16]. In a DENV-2 outbreak in 1997, secondary DENV-2 infections 18–20 years after the primary DENV-1 infection were clinically apparent, and 4.4% of these patients had severe infections [17]. Vaughn et al. (2000) reported that patients with DENV-2 infections experienced more severe disease than those infected with other serotypes, since most of the DENV-2 infections were secondary [18]. Disease severity in an area can be affected by the DENV serotypes that previously circulated there. A meta-analysis of more than 31 studies showed that DENV-3 isolates from Southeast Asia (SEA) were associated with the highest percentage of severe disease in primary infection, whereas DENV-2, DENV-3, and DENV-4 isolates from SEA, as well as DENV-2 and DENV-3 from non-SEA regions, exhibited the highest percentage of severe disease in secondary infection [19]. Although there were slight variations in the conclusions of the reports, monitoring the serotype of DENV is important when examining the epidemiology of dengue. Furthermore, the Guideline for the Prevention of Vector-borne Diseases published by the Ministry of Health, Labour, and Welfare, Japan (H27-No. 260) recommends that the serotype be determined in cases of dengue infection in Japan.

Serotype-specific detection of DENV in the early stages of infection is essential for adequate epidemiologic monitoring and for the development of strategies to reduce mortality. In addition, rapid and accurate differential diagnosis is required for prompt clinical management to reduce mortality. Rapid immunochromatography methods or lateral flow immunoassays are well suited for rapid-turnaround analyses without the need for specialized reagents, equipment, or trained personnel [20,21,22,23,24]. Rapid tests are generally low cost, can often be transported without refrigeration, and can be used in austere environments. Although nucleic acid amplification methods are highly specific, and have low limits of detection compared with immunochromatography strips, their disadvantages include a requirement for equipment powered by electricity/batteries, specialized reagents, highly trained personnel, and availability of cold storage for maintaining enzyme activity.

Compared with serological markers (i.e., anti-DENV IgM), DENV non-structural protein 1 (NS1) is a more useful diagnostic marker for the early detection of DENV because it can be detected in the serum of DENV-infected patients from the onset of symptoms. The level of NS1 circulating in the bloodstream of DENV-1-infected patients is estimated to range from 0.01 to 50 μg/mL [25]. Detection of soluble NS1 by immunochromatographic assays has become standard for dengue diagnosis because it allows early diagnosis and more effective patient management due to their convenience [26,27,28,29,30,31].

Although NS1 is a highly conserved glycoprotein with serotype-specific epitopes that enable differentiation of DENV serotypes [32], the only commonly used laboratory method currently available to determine DENV serotype is RT-PCR, which is the most sensitive and specific diagnostic tool [33]. None of the currently available commercial rapid tests distinguish DENV serotypes. In this report, we describe viral NS1 antigen–based rapid tests that use monoclonal antibody (MAb) pairs to detect and distinguish the four DENV serotypes.

## 2. Materials and Methods

### 2.1. Ethics Statement

Protocols regarding the use of clinical specimens in the present study were reviewed and approved by the Ethics Committee of the Faculty of Tropical Medicine, Mahidol University, Thailand (TMEC 19-051). The animal studies described herein were carried out in accordance with the recommendations of “The Guide for the Care and Use of Laboratory Animals” of Osaka University, Japan. The protocol was approved by the Committee on the Ethics of Animal Experiments of the Research Institute for Microbial Diseases, Osaka University, Japan (approval number H27-10-1). All procedures were performed using best efforts to minimize animal suffering.

### 2.2. Immunization Strategies and MAb Production

Four-week-old female BALB/c mice were used for immunizations. A total of 100 μL of B7 cells [34] (total of 2.5 × 10^6^ cells) infected with DENV was mixed with an equal volume of complete (for priming) or incomplete (for boosting) Freund’s adjuvant (FUJIFILM Wako Pure Chemical Corp., Osaka, Japan). Mice were prime-immunized intraperitoneally with DENV-infected B7 cells and then boosted at 2-week intervals with virion particles (3 × 10^4^ to 2 × 10^6^ focus-forming units (FFU)) or recombinant NS1 (100 µg) with incomplete Freund’s adjuvant until indirect immunofluorescence assays (IFAs) became positive for anti-dengue antibody in immunized mouse serum. Three days after the final booster, the mice were euthanized, and the spleen of each mouse was harvested and processed. Hybridomas were generated by fusing splenocytes from individual mice with PAI fusion partner cells, a process facilitated by polyethylene glycol 1500 (Roche Applied Sciences, Penzberg, Germany). To select hybridomas, fused cells were seeded in 96-well plates and maintained in DMEM supplemented with 15% fetal bovine serum and hypoxanthine-aminopterin-thymidine (Invitrogen, Carlsbad, CA, USA) for selection and hypoxanthine thymidine (Invitrogen) for maintenance. The culture medium was changed every 3 days for 2 weeks. Culture supernatants from successfully fused cells were subjected to primary screening using an IFA to select hybridomas secreting dengue-specific immunoglobulin.

### 2.3. IFA

MAbs were screened using an IFA. Briefly, 96-well plates containing Vero cells infected with DENV at multiplicity of infection of 0.5 were prepared. In case of capsid- or envelope-expressing cells, 100 ng of plasmid encoding mature capsid consisting of 100 amino acids of DENV-1 Mochizuki in pcDNA3 (pcD1C) or preM-E (pcD1ME, pcD2ME, pcD3ME, or pcD4ME) [35] was transfected into 293T cells using 0.4 μL of Lipofectamine 2000 according to the manufacturer’s instructions. At 48 h post-infection or post-transfection, the cells were washed three times with phosphate-buffered saline (PBS), fixed with formaldehyde (3.7% *v*/*v*, in PBS), and permeabilized with Triton X-100 (1% *v*/*v*, in PBS). After another three rounds of washing with PBS, 50 µL of culture supernatant was applied to the infected or transfected cells, and the plates were incubated at 37 °C for 1 h. The liquid was removed by aspiration, and the wells were washed three times with PBS to ensure that all unbound antibody was removed. Finally, 50 μL of secondary antibody (Alexa Fluor 488–conjugated goat-anti mouse IgG) was dispensed into each well, and the plates were incubated for 45 min at 37 °C and then subjected to a final washing step.

### 2.4. NS1 ELISA

A solution of diluted DENV NS1 antigen (The Native Antigen Company, Oxford, UK, 5 µg/mL) in PBS was added to the wells of microplates (Thermo Fisher Scientific, Waltham, MA, USA) and the plates were incubated at 4 °C overnight. The wells were then washed with PBS three times, incubated with blocking buffer (0.1% bovine serum albumin, 0.5% sucrose, 0.05% ProClin 300 in PBS) for 90 min at room temperature, and then hybridoma cell culture supernatant was added to the wells and incubated at 37 °C for 1 h. The wells were washed with PBS three times, and then a solution of diluted anti-mouse antibody conjugated with horseradish peroxidase (Millipore, Burlington, MA, USA) was added to each well and incubated at 37 °C for 1 h. The wells were washed with PBS three times, and then TMB substrate solution (KPL) was added and the wells were incubated at room temperature to allow for color development. HCl solution was added to each well to stop the reaction, and the absorbance at 450 nm was measured using a plate reader (Bio-Rad, Hercules, CA, USA).

### 2.5. MAb Isotype Determination

To determine the isotype of the heavy and light chains of MAbs, culture supernatant of each clone was tested using a commercially available mouse MAb isotyping kit (Isostrip; Roche Applied Sciences) according to the manufacturer’s protocol.

### 2.6. Immunochromatographic Assay Preparation

The IgG fraction purified from murine ascites fluid was used to develop an immunochromatographic test kit. The anti-DENV MAbs prepared in this study (Table 1) were immobilized onto a nitrocellulose membrane (10 μg/test) for the test line to capture NS1 protein. To prepare the control line, anti-mouse IgG (Immuno Probe) was immobilized onto a nitrocellulose membrane (6 μg/test) to capture mouse IgG. The detection antibodies (Table 1) were labeled with colloidal gold, impregnated onto glass fibers, dried, and placed at the conjugate pad between the test line and the sample-soaking region. The nitrocellulose membrane and glass fiber pad were assembled with a glass fiber sample pad on a plastic sheet. These assembled devices were stored in a bag with desiccant at room temperature until use.

### 2.7. Viruses

DENV-1 (Mochizuki strain), DENV-2 (16681), DENV-3 (H87), DENV-4 (H241), and 12 clinical isolates obtained from Thai patients (Supplemental Table S1) were propagated in C6/36 cells, and the virus titer in each culture supernatant was determined using real-time PCR [36]. The Japanese encephalitis virus (JEV) (Nakayama strain) and chikungunya virus (S27 strain) were prepared in Vero cells, while Sindbis virus (R68 strain) and Zika virus (MR766 strain) were prepared in BHK and C6/36 cells, respectively.

### 2.8. Clinical Sample Collection, Viral Load Measurement, and DENV Serotype Determination

Serum samples were collected from DENV-positive patients who presented to the Hospital for Tropical Diseases during 2018–2020 [37]. Samples were stored at −80 °C until further analysis. Viral RNA was extracted from 70 μL of clinical serum specimens using a QIAamp viral RNA mini kit (Qiagen, Hilden, Germany) according to the manufacturer’s instructions. The viral load data were quantified using One-step SYBR Green I–based quantitative RT-PCR specific for DENV, using previously described primer pairs and protocols [38]. Briefly, the final volume in each well was 20 μL, consisting of 10 μL of real-time master mix, 1 μL of probe/primer mix, 4 μL of nuclease-free water, and 5 μL of RNA template. The amplification condition was 5 min at 42 °C, 10 s at 95 °C, followed by 45 cycles of amplification for 5 s at 95 °C, 30 s at 55 °C, and 30 s at 72 °C, with melting curve analysis. A standard curve was used to determine the DENV RNA copy number based on the threshold cycles (Ct) on the CFX96 instrument (Bio-Rad). DENV-positive RNA samples were then analyzed according to the manufacturer’s instructions using a commercial dengue subtyping multiplex kit (Genesig, Chandler’s Ford, UK) for DENV serotype determination.

### 2.9. Antigen Detection Using the Immunochromatographic Devices

A total of 30 μL of serially diluted virus stock in MEM or DENV patient serum was mixed with 30 μL of specific buffer for each kit. The chromatographic stick was soaked in the mixture and incubated for 15 min at room temperature. The test band intensities were measured using a chromatogram reader (Hamamatsu Photonics, Bridgewater, NJ, USA) and expressed as the attenuation of reflected brightness in milli-absorbance units (mAbs). Bands with an intensity >15 mAbs were visible by eye. Schematic illustrations and photographs of the kits are shown in Supplementary Materials Figure S1. NS1 antigen detection devices from the SD-Bioline Duo kit (NS1Ag+IgG/IgM, The Alere Medical, Waltham, MA, USA) were used according to the manufacturer’s instructions.

## 3. Results

### 3.1. Generation of DENV-Specific MAbs

Mice were immunized with DENV-infected B7 cells and boosted several times with recombinant NS1 protein, as described in the Materials and Methods. The culture supernatant of each established clone was assessed by indirect immunofluorescence staining of DENV-infected Vero cells. The viral structural protein to which each antibody bound was determined by IFA of cells expressing PreM-E or Capsid following plasmid transfection. Reaction of the MAbs with NS1 proteins was confirmed using an in-house ELISA. Finally, we obtained 11 clones of DENV-1-specific, 9 DENV-2-specific, 4 DENV-3-specific, and 5 DENV-4-specific anti-NS1 clones. A total of 20 clones reacted with NS1 of all serotypes. It was initially planned to use a single MAb reacting to all four serotypes as a gold-labeled detection antibody in all of the serotyping kits to reduce the costs of kit preparation. However, after the preliminary screening of all the combinations of gold-labeled MAbs reacting to all of the serotypes and serotype-specific capture MAbs, it was found that most of the combinations provided only low signal-to-background ratios. Therefore the 8 clones listed in Table 1 were selected for assembly of the immunochromatographic devices. The reactivity of each MAb with the four serotypes of laboratory and clinical isolates of DENV and JEV was evaluated by IFA, as shown in Table 2. Two types of DENV-4-specific devices were assembled (Table 1).

### 3.2. Detection of Recombinant NS1 Proteins Using the Immunochromatographic Devices

**Figure 1:**
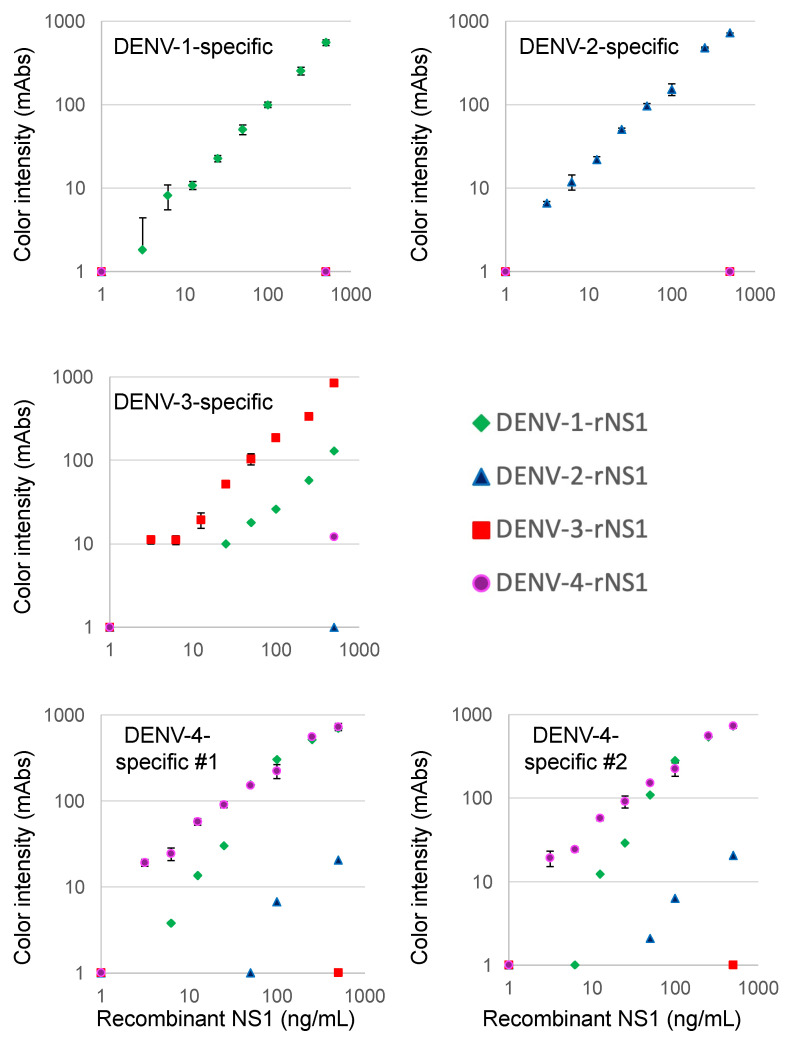
Evaluation of reactivity to recombinant NS1 proteins.

We first tested NS1 recombinant protein purchased from The Native Antigen Company in our novel immunochromatographic devices. As shown in Figure 1, the DENV-1-specific device detected 25.0 ng/mL of NS1 protein of DENV-1 (Nauru/WesternPacific/1974), but did not show any signal for 500 ng/mL of DENV-2 (Thailand/16681), DENV-3 (Sri Lanka D3/H/IMTSSA-SRI/2000/1266) or DENV-4 (Dominica/814669/1981) NS1 proteins. The DENV-2-specific device detected 12.5 ng/mL of NS1 protein of DENV-2, but did not show any signal for NS1 of the other serotypes. The DENV-3-specific device detected 6.3 ng/mL of NS1 protein of DENV-3, but did not show any signal for 500 ng/mL of DENV-2 or DENV-4 NS1. The DENV-3-specific device showed a signal for >50 ng/mL of the DENV-1 NS1 protein, but the signal for 500 ng/mL of the DENV-1 NS1 protein was lower than that of the DENV-3 NS1 protein. In the case of the DENV-4 #1-specific device, 3.1 ng/mL of the DENV-4 NS1 protein was detected. There was no signal for 500 ng/mL of the DENV-2 or DENV-3 NS1 protein, but the signal for the DENV-1 NS1 protein was similar to that of the DENV-4 NS1 protein. Virtually the same results as observed with the DENV-4 #1-specific device were obtained with the DENV-4 #2-specific device.

The limits of detection for the DENV serotype-specific devices were defined by chromatographing a dilution of recombinant NS1 protein (green diamonds, DENV-1; blue triangle, DENV-2; red squares, DENV-3; and purple circles, DENV-4) and then quantification of the signal color intensity (mAbs: milli-absorbance). When we used a corresponding serotype virus to each device, data are expressed as the mean and standard deviation of triplicate samples. When we used non-corresponding serotype viruses to each device, each of the data points represented the result of a single sample.

### 3.3. Detection of NS1 in Cultured Viruses Using the Immunochromatographic Devices

**Figure 2:**
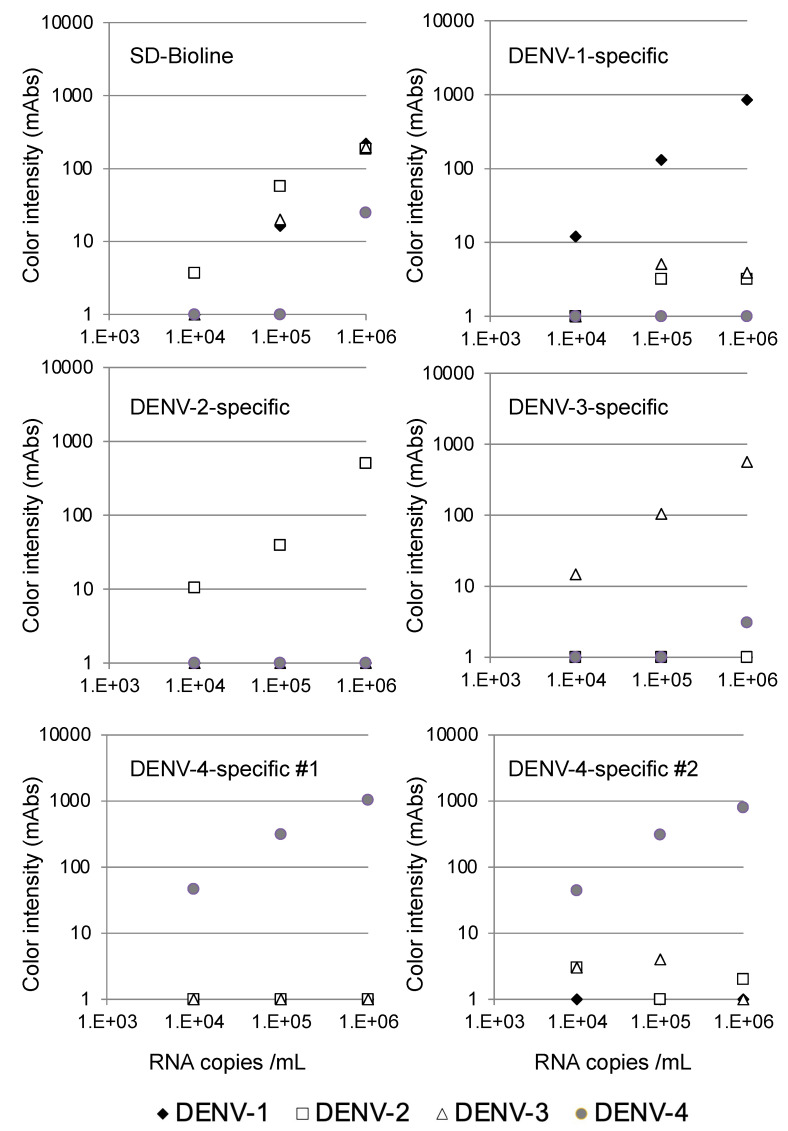
Evaluation of serotype specificity using laboratory strains.

The specificity of the serotype-specific devices was evaluated using all four DENV serotypes. As shown in Figure 2, the DENV-1-specific device clearly detected 10^5^ copies/mL of the DENV-1 strain Mochizuki, but the other DENV serotypes were not detected at 10^6^ copies/mL, indicating that the DENV-1-specific device detected DENV-1 specifically. Similarly, the DENV-2-specific device clearly detected 10^5^ copies/mL of the DENV-2 strain 16681, but the other three DENV serotypes were not detected at 10^6^ copies/mL, indicating that the DENV-2-specific device detected DENV-2 specifically. The DENV-3-specific device clearly detected 10^5^ copies/mL of the DENV-3 strain H87, but the other DENV serotypes were not detected at 10^6^ copies/mL, indicating that the DENV-3-specific device detected DENV-3 specifically. Both the DENV-4 #1 and #2-specific devices clearly detected 10^4^ copies/mL of the DENV-4 strain H241. Despite the apparent cross-reactivity of the DENV-4-specific devices with DENV-1 NS1, the other DENV serotypes, including DENV-1, were not detected at 10^6^ copies/mL, indicating that both DENV-4-specific devices detected DENV-4 specifically. On the other hand, the SD-Bioline Ag detection kit detected NS1 proteins in the culture supernatant at 10^5^ copies/mL of DENV-1, DENV-2, and DENV-3, but failed to detect DENV-4 at a similar concentration, indicating that the sensitivity of detection was lower than for the DENV-4-specific devices.

**Figure 3:**
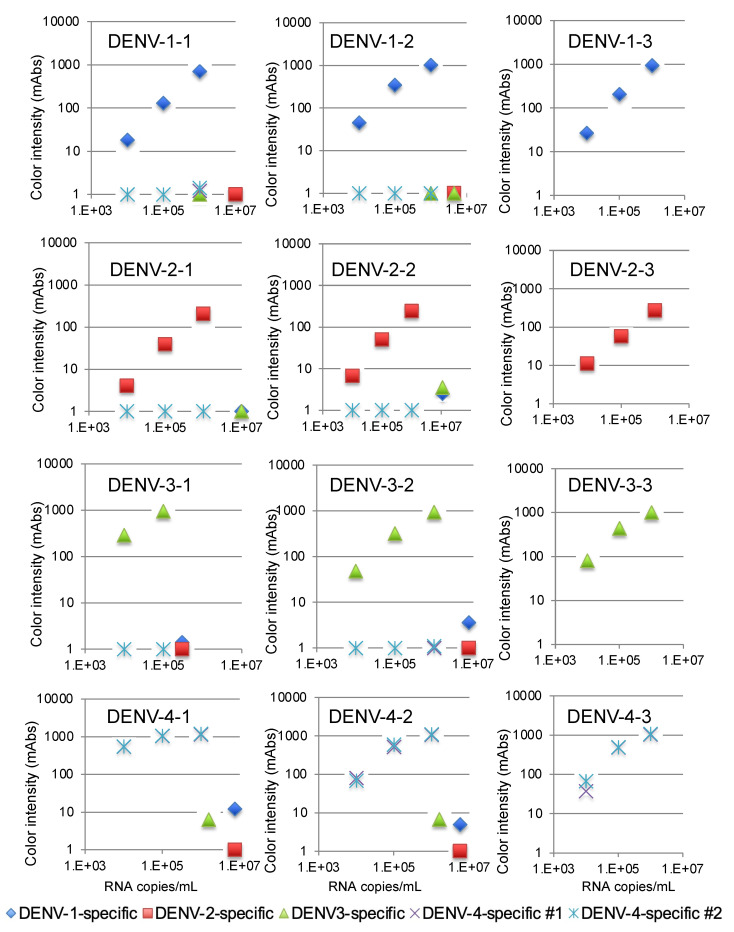
Evaluation of kit reactivity using clinical isolates from Thailand.

We then validated the devices using a recent isolate of the virus from Thailand. The variations in the NS1 amino acid sequence of these isolates are summarized in Supplementary Materials Table S1. The DENV-1-specific device detected 10^4^ copies/mL of three DENV-1 isolates, as shown in Figure 3. The DENV-2-specific device detected 10^5^ copies/mL of three DENV-2 isolates (Figure 3), and the DENV-3-specific device detected 10^4^ copies/mL of three DENV-3 isolates (Figure 3). Finally, the two DENV-4-specific devices detected 10^4^ copies/mL of three DENV-4 isolates (Figure 3). No cross-reactivity among the different serotypes was observed with these serotype-specific devices (Figure 3).

We also confirmed that the devices did not react with culture supernatant of chikungunya virus (S27 strain, 2.3 × 10^9^ PFU/mL), JEV (Nakayama strain, 1.9 × 10^9^ FFU/mL), Sindbis virus (R68, 6.4 × 10^9^ plaque-forming units (PFU)/mL), or Zika virus (MR766, 1 × 10^7^ PFU/mL) (Supplementary Table S3). On the other hand, the SD-Bioline Ag detection kit showed cross-reactivity with the JEV and Zika virus.

The limits of detection for DENV serotype-specific devices and SD Bioline were defined by chromatographing dilutions of viruses (black diamonds, Mochizuki strain of DENV-1; white squares, 16681 strain of DENV-2; white triangles, H89 strain of DENV-3; and gray circles, H241 strain of DENV-4) with known titers (10^4^, 10^5^, and 10^6^ copies/mL) and then quantifying the signal color intensity (mAbs: milli-absorbance). Each of the data points represents the result of a single device.

The limits of detection for the DENV-1-specific (blue diamonds), DENV-2-specific (red squares), DENV-3-specific (green triangles), and DENV-4 #1 and #2-specific devices (purple crosses and blue asterisks) were defined by chromatographing dilutions of viruses with known titers (10^5^, 10^6^, and 10^7^ copies/mL) and then quantifying the signal color intensity (mAbs: milli-absorbance). Each of the data points represents the result of a single device.

### 3.4. Detection of NS1 in Clinical DENV Specimens

**Figure 4:**
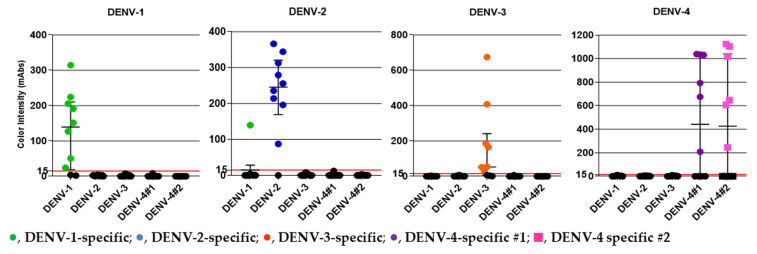
DENV patient serum samples from the Hospital for Tropical Diseases, Bangkok, Thailand, collected during 2018–2020, were used in the study. DENV serotype of the specimen is shown at the top of each panel. The y-axis indicates color intensity quantified as milli-absorbance (mAbs) units using an immunochromatogram reader. Results obtained from five serotype-specific devices are shown. The green (●), blue (●), orange (●), and purple (●) circles and pink (■) squares represent positive serum samples detected using the DENV-1, -2, -3, -4 #1, and -4 #2 devices, respectively. Median and interquartile ranges of color intensity (mAbs) are shown. The grid line at 15 mAbs on the y-axis was set as the cut-off value of the devices. Each of the data points represents the result of a single device.

We obtained serum samples from 40 DENV-positive patients (confirmed by RT-PCR) from the Hospital for Tropical Diseases during 2018–2020 [32]. The samples included 10 DENV-1, 10 DENV-2, 10 DENV-3, and 10 DENV-4 specimens. The median Ct values for the DENV-1, DENV-2, DENV-3, and DENV-4 specimens were 18.24, 18.64, 24.24, and 23.46, respectively, which corresponded to 9.36 × 10^5^, 8.66 × 10^5^, 3.76 × 10^4^, and 9.40 × 10^4^ PFU/mL, respectively. Table 3 and Figure 4 summarize the clinical evaluation of the DENV serotype-specific devices. All of the DENV serotype-specific devices except the DENV-1-specific device exhibited 100% specificity; a false-positive result for one DENV-2 specimen was obtained with the DENV-1-specific device. The overall agreement (%) was >90% for all devices except the DEV-4 #2-specific device, which showed an overall agreement of 89.7%. Specimen ID numbers, DENV serotype, genotype, and other data are shown in Supplementary Table S2.

## 4. Discussion

We successfully developed serotype-specific rapid detection systems for DENV NS1. The introduction of a different DENV serotype into a region could cause outbreaks involving severe hemorrhagic fever because infection with one particular serotype does not provide long-lasting cross-protection, and heterologous serotype infections lead to increased prevalence of hemorrhagic fever in dengue. Serotype-specific detection has implications regarding anti-DENV drug development. For example, in treatment of influenza virus infection, amantadine does not exhibit equal efficacy against influenza virus type B and influenza virus type A. Therefore, in the future, knowledge regarding circulating serotypes could affect treatment of DENV infection outbreaks if serotype-specific anti-DENV drugs are developed. Our novel devices reported here would accelerate both DENV diagnosis and epidemiologic research in DENV-endemic areas.

Numerous reports have shown that the DENV serotype is related to disease severity, although the conclusions of these reports were not consistent. In addition, a neutralizing antibody titer of 1:21 to 1:80 is reportedly a risk factor for severe dengue [39]. The availability of rapid serotyping tests could accelerate research into characterizing worldwide serotype distribution and facilitate continuous analysis of DENV serotypes in each area, which could enhance our understanding of the relationship between serotype and disease severity.

Using recombinant NS1 protein, we observed cross-reactivity between the DENV-4-specific device and the DENV-1 NS1 protein (Figure 1). In contrast, when we examined the culture supernatant of isolated virus, no cross-reactivity between the DENV-4-specific device and DENV-1 was observed. The recombinant NS1 protein of DENV-1 was derived from genotype IV, whereas the viruses used in the present study were all genotype I. The amino acid sequence similarity to DENV-4 NS1 of the DENV-1 genotype IV NS1 was not specified (Table S1). The sensitivity and cross-reactivity should be carefully evaluated before these devices are used in areas in which DENV-1 genotype IV is circulating, such as the Western Pacific countries. It should also be noted that a single DENV-2 specimen, TM19-13, exhibited cross-reactivity with the DENV-1-specific device. Sequence analysis of DENV-2 NS1 from this specimen showed that the amino acid residues at positions 181 and 343 were different from NS1 of other DENV-2 isolates examined in the present study (Table S1). It will be necessary to determine whether these amino acid differences were associated with the observed cross-reactivity. The prevalence of these amino acid sequence differences in various geographical areas should also be determined. Previous studies showed variations in sensitivity and specificity depending on the collection date after the onset of illness, viral characteristics, and heterotypic DENV infection in NS1 DENV-specific detection using clinical specimens [40,41,42,43,44]. These factors might have also played a role in the cross-reactivity observed in the present study.

Commercially available NS1 antigen tests manufactured by Bio-Rad (Platelia) and Panbio (Dengue Early) have low sensitivity for detecting DENV-4 infections [28,45,46,47]. The aforementioned studies indicated the need for development of new NS1 antigen detection tests with improved sensitivity for DENV-4. As shown in Figure 2, the SD-Bioline Ag detection kit failed to detect NS1 protein in the culture supernatant at 10^5^ copies/mL of DENV-4, whereas the SD-Bioline kit detected DENV-1, DENV-2, and DENV-3 at the same dilution. Both of our DENV-4-specific devices detected NS1 protein in the culture supernatant at 10^4^ copies/mL of DENV-4.

Roltgen et al. (2018) developed an ELISA capable of distinguishing four DENV serotypes [48]. Similar ELISAs were also developed by other groups [40,41]. ELISAs have advantages in quantification, but immunochromatographic tests have the advantage of convenience for individual tests of a small number of samples [33]. Bosch et al. (2017) reported a rapid immunochromatographic test capable of distinguishing DENV serotypes using anti-NS1 antibodies, similar to our system [49]. Their detection limit was comparable to ours (4–20 ng/mL). Theirs had an advantage in being able to detect the Zika virus, whereas our devices would provide more rapid results, since their device needed the addition of four different gold-conjugated antibodies each time and took 15 min to 1 h to obtain results. Even though the sensitivity of our DENV serotype-specific devices was less than that of molecular detection by PCR, the gold standard of DENV serotyping, our DENV serotype-specific devices are much faster than molecular detection. Moreover, the presence of IgM and/or IgG does not affect the performance of our DENV serotype-specific devices.

Further research using clinical specimens from various geographical areas harboring different DENV genotypes will be required to maximize the sensitivity and specificity of our systems. Whole-blood assays should be developed for point-of-care devices. The inability to detect the Zika virus should also be addressed in the future so that our devices can be used in clinics in Zika-endemic areas.

## Figures and Tables

**Table 1:** Antibodies used in the immunochromatographic devices.

Device	Detection Antibody	Capture Antibody
	(Gold Label)	(Membrane Bound)
DENV-1-specific	A	E
DENV-2-specific	A	F
DENV-3-specific	D	G
DENV-4-specific #1	C	H
DENV-4-specific #2	B	H

**Table 2:** Reaction of antibodies in indirect immunofluorescence assay of virus-infected cells.

Antibody	Category	Subclass	Laboratory Strain					Clinical Isolate				Un-Infected
			DV1a	DV2b	DV3c	DV4d	JEVe	DV1f	DV2g	DV3h	DV4i	Vero
A	DENV-All	IgG1. kappa	++	++	+	++	(−)	ND	++	++	ND	(−)
B	DENV-All	IgG1. kappa	+	+	+	+	B	+	+	+	+	(−)
C	DENV-All	IgG1. kappa	+	+	+	+	B	+	+	+	+	ND
D	DENV-All	IgG1. kappa	+	+	B	+	(−)	ND	ND	ND	ND	(−)
E	DENV-1-specific	IgG1. kappa	+	(−)	(−)	(−)	ND	+	(−)	(−)	ND	ND
F	DENV-2-specific	IgG1. kappa	B	++	(−)	(−)	(−)	ND	++	(−)	ND	ND
G	DENV-3-specific	IgG1. kappa	B	B	+	(−)	B	B	B	+	B	(−)
H	DENV-4-specific	IgG2a. Kappa	(−)	(−)	(−)	+	ND	B	(−)	(−)	+	(−)

a, Mochizuki; b, 16681; c, H87; d, H241; e, Nakayama; f, DENV-1-1; g, DENV-2-3; h, DENV-3-1; i, DENV-4-1. B, high background; +, positive; ++, strong positive; (−), negative; ND, not done.

**Table 3:** Evaluation of DENV serotype-specific devices in DENV-positive clinical serum samples from the Hospital for Tropical Diseases, Bangkok, Thailand, during 2018–2020.

Device	DENV-1-Specific	DENV-2-Specific	DENV-3-Specific	DENV-4 #1-Specific	DENV-4 #2-Specific
Median color intensity (mAbs)	139.3	245.8	52.8	441.9	426.2
Sensitivity (%)	80.0	90.0	70.0	60.0	60.0
Specificity (%)	96.7	100.0	100.0	100.0	100.0
Overall agreement (%)	92.5	97.5	92.5	90	89.7 *

* Overall agreement (%) was 100 (6+29)/39 = 89.7, as one serum (PW23) was not evaluated with the DENV-4-specific #2 device.

## Data Availability

Data are contained within the article or Supplementary Material.

## Supplementary Materials

The following are available online at https://www.mdpi.com/article/10.3390/s21237809/s1. Table S1. NS1 protein amino acid sequences, Clinical specimens used to evaluate the dengue serotype-specific devices; Table S2. Clinical specimens used to evaluate the dengue serotype-specific devices; Table S3. Lack of cross-reaction of serotyping devices with other arboviruses; Figure S1. The principle of immunochromatographic devices for detection of NS1 protein.
