# Supplementary material for: Development of a Dengue Virus Serotype-Specific Non-Structural Protein 1 Capture Immunochromatography Method
Source: Sensors (Basel). 2021 Nov 24;21(23):7809. doi: 10.3390/s21237809 (PMC8659457; doi:10.3390/s21237809)
Supplement: Supplementary file 1 [file sensors-21-07809-s001.zip › sensors-1449645-supplementary/Figure S1.pdf]

sensors

Supplementary Figure

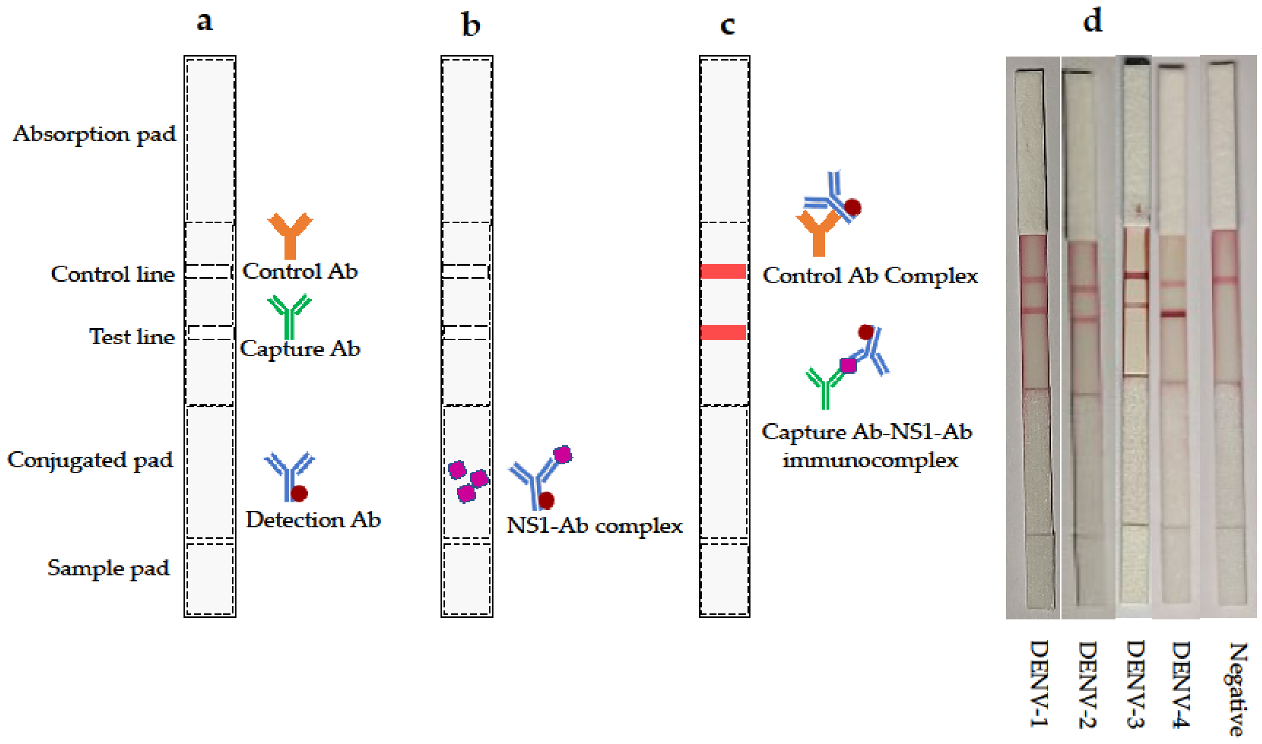

Figure S1. The principle of immunochromatographic devices for detection of NS1 protein. The detection antibodies were conjugated with colloidal gold nanoparticles (red circles), and capture antibodies were fixed on membrane (a). The NS1 protein (red squares) were captured with the conjugated antibody as antigen-antibody complexes (b). After that, these complexes flowed through and bound with the capture antibody at the test line (c). Appearance of color at test and control lines indicated a positive result. Photographs of developed strips are shown (d).The detection and capture antibodies of DENV serotype-specific devices are described in Table 1.
