# Supplementary material for: Development of a Dengue Virus Serotype-Specific Non-Structural Protein 1 Capture Immunochromatography Method
Source: Sensors (Basel). 2021 Nov 24;21(23):7809. doi: 10.3390/s21237809 (PMC8659457; doi:10.3390/s21237809)
Supplement: Supplementary file 1 [file sensors-21-07809-s001.zip › sensors-1449645-supplementary/Tables S2 and S3.pdf]

sensors MDPI

Supplementary Table

Table S2. Clinical specimens used to evaluate the dengue serotype-specific devices.

Sample ID	Collection date	Ct value	PFU/ml	serotype-Genotype	Clade	DENV IgM	DENV IgG	Intensity of color (mAbs)				
								DENV-1	DENV-2	DENV-3	DENV-4	DENV-4B
DV1I-TM18-20	28-May-18	18.00	1.39E+06	1-I	Ia	NA	NA	151.3	0.0	4.5	8.2	0.0
DV1I-TM19-70	23-Dec-19	15.38	3.99E+06	1-I	Ib	NA	NA	191.0	2.8	0	0.0	0.0
DV1I-TM19-40	29-Nov-19	15.79	3.92E+06	1-I	Ib	NA	NA	224.2	2.4	0	0.0	0.0
DV1I-TM19-52	10-Dec-19	19.06	5.19E+05	1-I	Id	NA	NA	205.8	0.0	0	0.0	0.0
DV1I-TM19-75	28-Dec-19	19.29	4.56E+05	1-I	Ia	NA	NA	314.4	3.0	0	0.0	0.0
DV1I-TM19-24	22-Nov-19	20.04	2.73E+05	1-I	Ic	NA	NA	50.6	2.6	2.6	0.0	0.0
DV1I-TM20-21	16-Feb-20	17.24	1.66E+06	1-I	Ib	Negative	Negative	2.0	3.3	0	0.0	0.0
DV1I-TM20-47	19-Aug-20	17.89	1.11E+06	1-I	Ib	NA	NA	127.2	2.2	8.1	0.0	0.0
DV1I-TM20-24	27-Jun-20	18.49	7.66E+05	1-I	Ib	NA	NA	3.8	4.5	0	0.0	0.0
DV1I-TM20-25	5-Jul-20	19.95	3.10E+05	1-I	Id	NA	NA	24.1	0.0	4.3	2.3	0.0
DV2C-TM19-37	28-Nov-19	15.84	3.79E+06	2-C	Cb	NA	NA	0.0	255.8	5.2	12.3	0.0
DV2A-TM19-13	19-Nov-19	17.7	1.18E+06	2-AI	AIb	NA	NA	139.8	279.2	0.0	0.0	0.0
DV2C-TM19-41	30-Nov-19	18.67	6.42E+05	2-C	Cc	NA	NA	0.0	312.9	0.0	0.0	0.0
DV2C-TM19-32	26-Nov-19	19.23	4.52E+05	2-C	Cc	NA	NA	3.7	87.7	0.0	0.0	3.0
DV2C-TM19-31	26-Nov-19	19.68	3.40E+05	2-C	Cc	NA	NA	1.3	196.3	3.2	0.0	0.0
DV2A-TM19-43	3-Dec-19	22.74	6.72E+04	2-AI	AIb	NA	NA	0.0	214.2	0.0	0.0	1.2
DV2A-TM20-96	3-Nov-20	16.57	3.39E+06	2-AI	AIa	NA	NA	1.5	344.3	0.0	0.0	0.0
DV2A-TM20-94	30-Oct-20	18.88	1.02E+06	2-AI	AIb	NA	NA	0.0	0.0	0.0	1.8	0.0
DV2C-TM20-65	22-Sep-20	18.14	1.50E+06	2-C	Cb	NA	NA	0.0	235.7	0.0	0.0	0.0
DV2C-TM20-36	3-Aug-20	18.61	7.10E+05	2-C	Ca	NA	NA	0.0	366.3	8.2	0.0	0.0
DV3I-TM18-31	26-Jun-18	25.03	3.94E+04	3-I	NA	Negative	Positive	0.0	0.0	54.3	6.2	0.0

DV3I-TM18-22	4-Jun-18	28.83	5.71E+03	3-I	NA	Negative	Negative	3.1	6.3	674.9	0.0	0.0
DV3III-TM18-12	8-May-18	23.03	1.09E+05	3-III	NA	NA	NA	0.0	0.0	164.8	0.0	0.0
DV3I-TM18-34	7-Jul-18	28.28	7.57E+03	3-I	NA	Negative	Negative	0.0	2.5	0.9	0.0	0.0
DV3-PW26	18-Oct-18	22.14	4.16E+04	NA	NA	NA	NA	0.0	0.0	3.1	0.0	0.0
DV3I-PW23	10-Oct-18	37.13	3.15E+00	3-I	NA	NA	NA	0.0	4.9	407.8	0.0	0.0
DV3I-TM20-10	11-Jan-20	20.02	2.98E+05	3-I	NA	NA	NA	0.0	0.0	186.1	0.0	0.0
DV3I-TM20-02	2-Jan-20	21.44	1.24E+05	3-I	NA	NA	NA	0.0	0.0	51.3	0.0	0.0
DV3III-TM20-07	10-Jan-20	23.45	3.58E+04	3-III	NA	NA	NA	0.0	0.0	24.6	0.0	0.0
DV3I-TM20-09	11-Jan-20	27.05	3.86E+03	3-I	NA	NA	NA	0.0	0.0	6.5	0.0	0.0
DV4I-TM20-76	3-Oct-20	28.8	2.34E+03	4-I	Ic	NA	NA	7.0	0.0	6.1	0.0	0.0
DV4I-TM18-7	27-Mar-18	22.82	1.20E+05	4-I	Ia	NA	NA	10.5	0.0	0.0	0.0	0.0
DV4I-TM20-90	21-Oct-20	19.21	8.60E+05	4-I	Ic	Negative	Negative	0.0	0.0	6.8	1034.6	1103.7
TM-DV4-20-41	14-Aug-20	19.54	4.00E+05	4-I	NA	Negative	Positive	5.1	0.0	0.0	1030.1	1123.8
TM-DV4-20-69	28-Sep-20	19.61	6.99E+05	4-I	NA	NA	NA	0.0	0.0	0.0	1038.9	1018.0
DV4I-TM20-64	18-Sep-20	20.03	5.63E+05	4-I	Ic	Negative	Positive	3.7	4.0	0.0	0.0	0.0
DV4I-TM20-59	11-Sep-20	24.11	6.76E+04	4-I	Ib	NA	NA	0.0	3.6	0.0	0.0	0.0
DV4I-TM20-98	9-Nov-20	26.01	2.52E+04	4-I	Ic	Negative	Positive	0.0	3.2	0.0	674.9	607.2
DV4I-TM20-53	29-Aug-20	26.27	2.20E+04	4-I	Ic	Positive	Positive	0.0	4.1	3.4	792.8	646.9
DV4I-TM20-5	9-Jan-20	27.23	3.46E+03	4-I	Ib	NA	NA	2.9	3.2	8.8	208.8	245.1
C Cosmopolitan												
AI Asian I												

sensors MDPI

Table S3. Lack of cross reaction of serotyping devices with other arboviruses.

Device	Intensity of color (mAbs)			
	JEV (Nakayama) 1.9 x 10^9 FFU/mL	Sindbis virus (R68) 6.4 x 10^9 PFU/mL	CHIKV(S27) 2.3 x 10^9 PFU/mL	Zika virus (MR766) 1 x 10^7 PFU/mL
DENV-1-specific	0	0	0	0
DENV-2-specific	0	0	0	0
DENV-3-specific	0	7.3	13.5	0
DENV-4-specific #1	0	0	0	0
DENV-4-specific #2	0	0	0	0
SD Bioline	128.6	ND	ND	77.5

JEV, Japanese encephalitis virus; CHIKV, chikungunya virus; ND, not done
